# Inactivation of Chikungunya virus by 1,5 iodonapthyl azide

**DOI:** 10.1186/1743-422X-9-301

**Published:** 2012-12-04

**Authors:** Anuj Sharma, Paridhi Gupta, Radha K Maheshwari

**Affiliations:** 1 Department of Pathology, Uniformed Services University of the Health Sciences, 4301 Jones Bridge Road, Bethesda, MD 20814, USA; 2 Department of Biological Sciences, Birla Institute of Technology and Science, Pilani, Rajasthan, India

**Keywords:** Chikungunya virus, Inactivated vaccine, Iodonapthyl azide

## Abstract

**Background:**

Chikungunya virus (CHIKV) is an arthropod borne alphavirus of the family *Togaviridae*. CHIKV is a reemerging virus for which there is no safe prophylactic vaccine. A live attenuated strain of CHIKV, CHIK181/25, was previously demonstrated to be highly immunogenic in humans, however, it showed residual virulence causing transient arthralgia.

**Findings:**

In this study, we demonstrate the complete inactivation of CHIKV181/25 by 1,5 iodonapthyl azide (INA). No cytopathic effect and virus replication was observed in cells infected with the INA-inactivated CHIKV. However, a reduction in the INA-inactivated CHIK virus-antibody binding capacity was observed by western blot analysis.

**Conclusion:**

INA completely inactivated CHIKV and can further be explored for developing an inactivated-CHIKV vaccine.

## Findings

Earlier we have shown that 1,5 iodonapthyl azide (INA) and UV irradiation completely inactivated the Venezuelan equine encephalitis virus (VEEV) [1,2]. In this study, we have extended that approach to evaluate the inactivation of Chikungunya virus (CHIKV). CHIKV is an arthropod borne alphavirus of the family *Togaviridae*. It is a reemerging virus that has caused wide spread outbreak in the nations surrounding the Indian Ocean and Africa since 2004 [3]. Among the alphaviruses, CHIKV is one of the most important human pathogens due to the frequent outbreaks worldwide, causing large scale morbidity and economic losses. CHIKV infection has been reported in the travelers returning from the epidemic regions, thereby indicating a potential of dissemination to the previously known naïve regions for CHIKV [4]. Further, E1-A226V mutation in the CHIKV strain of 2005–2006 outbreak has increased the susceptibility of *Aedes albopictus*, which is endemic to the previously CHIKV naïve regions [5,6]. Therefore, there is an ever increasing risk of CHIKV outbreak in the areas such as the United States of America, where CHIKV is not endemic. Such potential outbreak may be devastating due to lack of immunity to CHIKV in the endemic population. There are no specific drugs against CHIKV and patients are symptomatically treated with non steroid anti-inflammatory drugs [7]. In the absence of antiviral drugs, preventive measures that include effective CHIKV vaccine are urgently needed. The United States Army had developed and tested a live attenuated strain of CHIKV, CHIKV181/25 for vaccine application. CHIKV181/25 demonstrated an excellent immunogenic profile (98% seroconversion), however, transient arthralgia was observed in about 8% of vaccine recipients [8]. Several other approaches such as chimeric CHIKV on other alphavirus backbone, CHIK virus like particles, formalin inactivated CHIKV, DNA immunization and passive immunization with human polyvalent immunoglobulin have also been tested [9-13].

INA is a photoactive hydrophobic azide molecule that sequesters in the hydrophobic domain of the biomembranes and binds to the membrane proteins upon irradiation with long wavelength ultra-violet (UV) light. It was initially used for labeling the biological membranes [14]. Interaction of INA with proteins inactivates the membrane protein while conserving the ectodomain protruding outside the membrane [15]. This property of INA has been used to inactivate influenza virus, Ebola virus, VEEV, vaccinia virus, pixuna virus, simian and human immunodeficiency viruses and provide potential vaccine candidates [1,2,15-18]. In this study, we have evaluated the dose dependent inactivation of CHIKV181/25 by INA.

**Figure 1:**
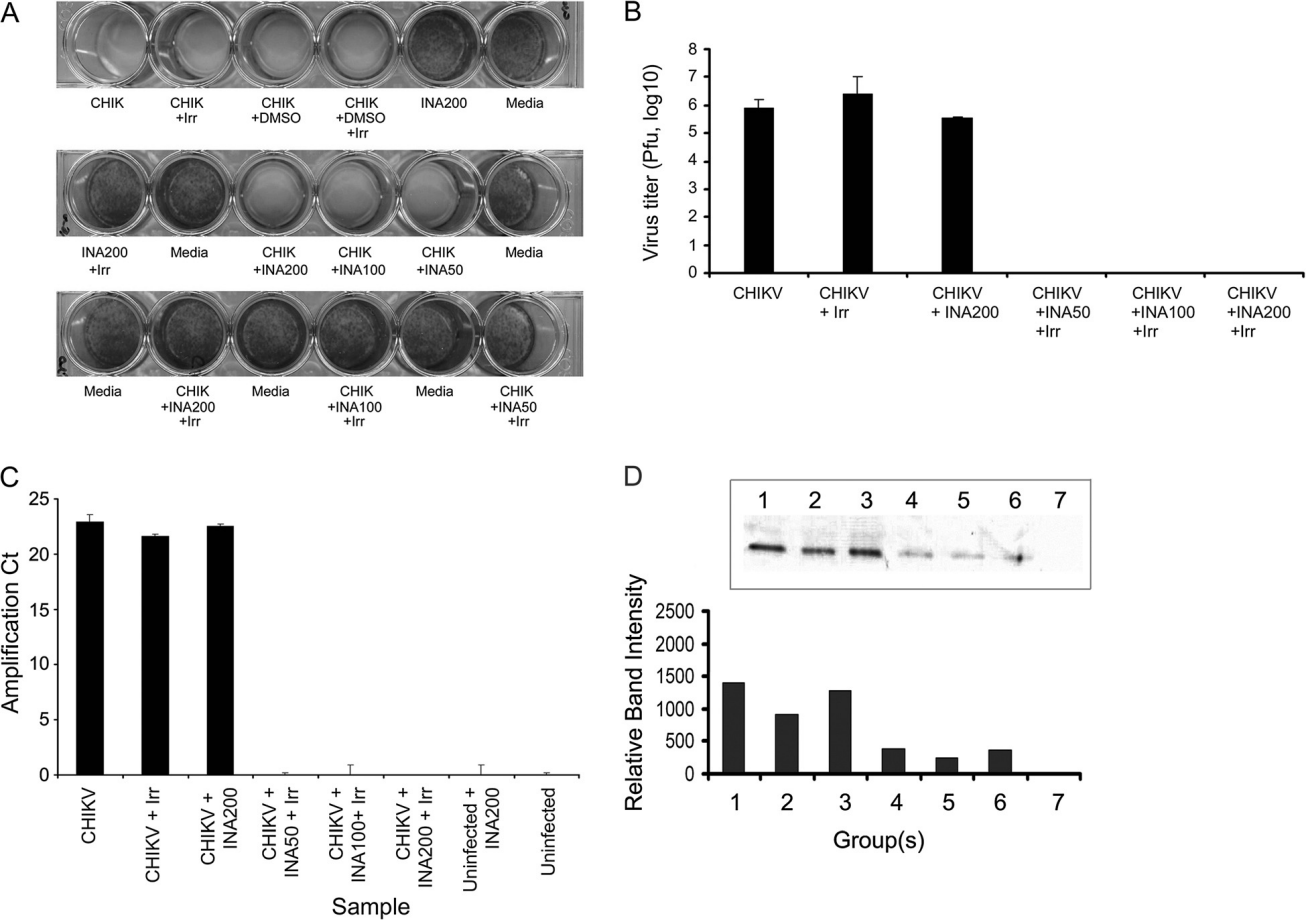
**Inactivation of CHIKV by INA: Inactivation of CHIKV by INA was evaluated as CPE, virus titer and viral RNA transcripts.A**: BHK cells were infected with various test and control samples and virus induced CPE was observed. Cells were stained with 0.1% crystal violet dye in 4% neutral buffered formalin. No CPE was observed in cells infected with INA treated CHIKV in combination with UV irradiation and cells replicated normally as evident by the stain uptake by the cells similar to control cells without virus infection. Irradiation of CHIKV alone and treatment with DMSO (since INA stocks were made in DMSO) did not affect the infectivity as evident by the loss of cells in these wells. **B**: Virus titer was measured in the supernatant of the cells infected with non-inactivated and INA-inactivated CHIKV. No virus was detected in the supernatant of INA-inactivated CHIKV and treatment with INA alone did not affect the virus replication. **C**: Viral transcripts were detected in the supernatants of cells infected with the non-inactivated and INA-inactivated CHIKV. No viral transcripts were detected in the cells infected with CHIKV inactivated with 50, 100 and 200 μM dose of INA in combination with UV irradiation. **D**: Western Blot analysis of the CHIKV proteins showed low binding of anti-CHIKV polyclonal antibody to CHIKV upon inactivation with INA. 1 = CHIKV; 2 = CHIKV + UV Irradiation; 3 = CHIKV + INA (200 μM); 4 = CHIKV + INA (50 μM) + UV Irradiation; 5 = CHIKV + INA (100 μM) + UV Irradiation; 6 = CHIKV + INA(200 μM) + UV Irradiation; and 7 = INA (200 μM) alone. Irr = UV irradiation.

**Figure 2:**
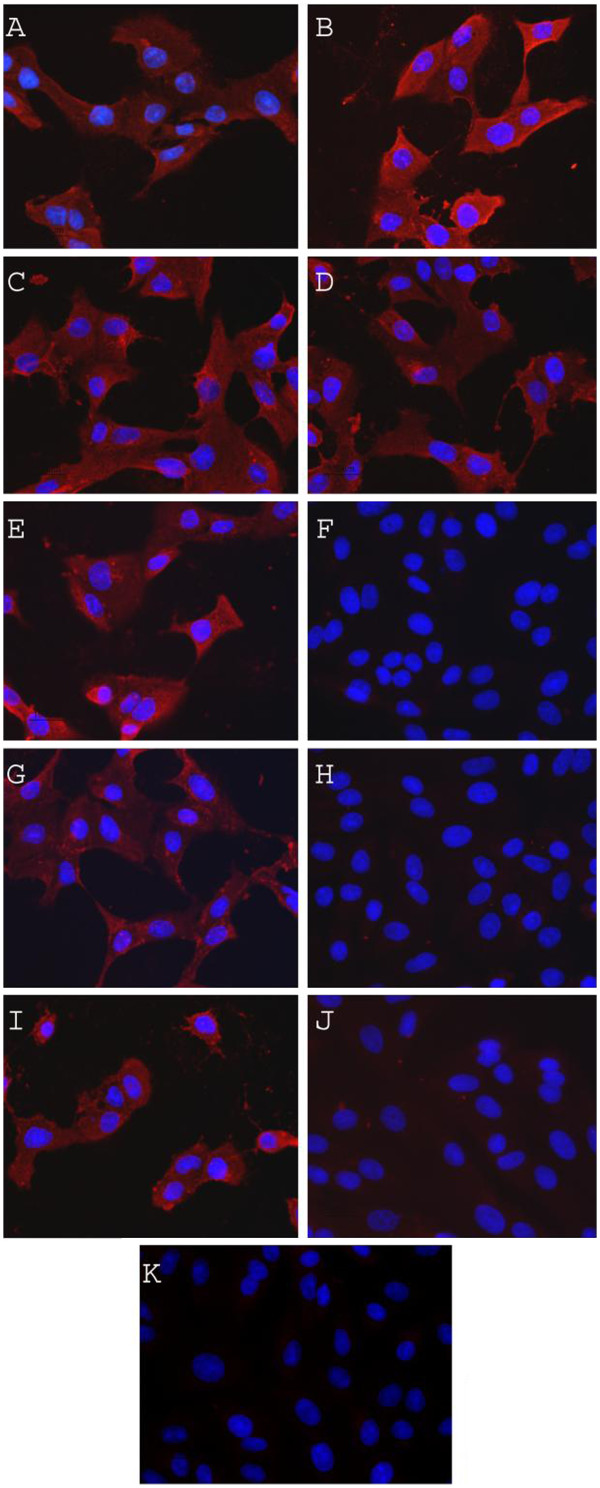
**Evaluation of CHIKV inactivation by immunofluorescence: BHK cells were infected with non-inactivated and INA-inactivated CHIKV and CHIKV specific immunofluorescence was evaluated as described in the text.A**: CHIKV; **B**: CHIKV + UV Irradiation; **C**: CHIKV + DMSO; **D**: CHIKV + DMSO + UV Irradiation; **E**: CHIKV + INA (50 μM); **F**: CHIKV + INA (50 μM) + UV Irradiation; **G**: CHIKV + INA (100 μM), **H**: CHIKV + INA (100 μM) + UV Irradiation; **I**: CHIKV + INA (200 μM); **J**: CHIKV + INA (200 μM) + UV Irradiation; **K**: uninfected.

CHIKV181/25 was kindly provided by Drs. Parker MD and Glass PJ of the United States Army Medical Research Institute (USAMRIID), Frederick, MD. INA was obtained from Dr. Blumenthal RM, NCI, Frederick under a material transfer agreement. 0.5 mg of sucrose gradient purified CHIKV was diluted with 1X DPBS and vortexed to mix and disintegrate the virus clusters. 20 mM INA was then added to make a final concentration of INA to 50, 100 and 200 μM. The final volume of the reaction was set at 1.0 ml. Virus + INA suspension was then irradiated with UV radiation for a total of 5 min as described before [1,2]. Samples were irradiated with long wavelength UV radiation. A glass filter was used to filter out the short wavelength UV radiation [1]. Samples were then immediately stored at -80°C until further use. Preliminary screening of virus inactivation was done by infecting BHK cells with a multiplicity of infection (MOI) of 1. Cells were observed for virus induced cytopathic effect (CPE) such as rounding and sloughing off from the surface and were stained with 0.1% crystal violet solution in 4% neutral buffered formalin. In our previous study, we have used glutathione to quench the free INA that may be present in the virus suspension [1]. However, in this study, addition of glutathione alone to the virus suspension resulted in an inconsistent non-specific inhibition of CHIKV181/25 infectivity (data not shown). Therefore, glutathione was omitted in the final inactivation experiments in the present study. Replication of INA-inactivated CHIKV in BHK cells was also evaluated by CHIKV specific immunofluorescence. Briefly, cells were infected with MOI of 1 and at 48 hr post infection fixed with 1:1 acetone: methanol for 10 min at room temperature. Slides were blocked with 1% BSA for 45 min at room temperature followed by incubation with primary antibody (1:200, CHIKV hyper-immune mouse ascetic sera, Catalog#VR64, ATCC, Manassas, VA) for 1 hr at 37°C. Slides were then washed and secondary antibody (Rhodamine labeled goat anti-mouse IgG) was added and incubated for 30 min at 37°C. Slides were washed and mounted using mounting media containing DAPI (Vectastain, Vector Labs Inc). Virus titer in the cell supernatants was measured by the standard plaque assay. Viral transcripts in the supernatant from the infected BHK cells were analyzed by real-time RT-PCR. The primer set used for the PCR was following; forward primer: 5-GCCAGACACGGAGACGCCAAC-3 and reverse primer: 5-TGACCCTACTGAGAACAGCAC-3. The primer set was targeted against the non-structural protein 4 gene of CHIKV181/25 with expected amplicon size of 340 bp. Western blot analysis was done to test the antibody binding capacity of INA-inactivated CHIKV using a polyclonal antibody against CHIKV. Briefly, 12.5 μg of protein for each sample was separated on 4-20% novex tris-glycine gel (Invitrogen Inc., Carlsbad VA). Protein bands were transferred on nitrocellulose membrane (Amhersham Biosciences UK Limited) at 125 mA overnight at 4°C. Viral proteins were detected by standard western blot procedure using polyclonal antibody (1:200) against CHIKV (CHIKV hyper-immune mouse ascitic sera, Catalog#VR64, ATCC, Manassas, VA) and alkaline phosphatase conjugated secondary antibody (1:25000 in 1XTBST, goat anti-mouse IgG, Catalog# AP124A, Chemicon International). Blot was developed using 10 ml substrate for alkaline phosphatase (Catalog# S3841, Promega Inc., Madison, WI) at room temperature for 15 min. A complete inactivation of CHIKV by INA was achieved in virus samples that were treated with INA in combination with UV irradiation. This group will now be addressed as INA-inactivated CHIKV through the rest of manuscript. No CPE was observed in the cells infected with INA-inactivated CHIKV (Figure 1A). Immunofluorescence staining for CHIKV also showed no viral antigen in the cells infected with INA-inactivated CHIKV whereas cells infected with non-inactivated CHIKV were positively stained for CHIKV antigen (Figure 2). Uninfected cells served as negative controls for CHIKV staining and infection. Virus titration by standard plaque assay [2] showed complete absence of CHIKV in supernatants from the cells infected with INA-inactivated CHIKV whereas significant viral titers were measured in the supernatants of cells infected with non-inactivated CHIKV (Figure 1B). Further, no viral transcripts were detected in the supernatant of cells infected with the CHIKV that was inactivated with 50, 100 and 200 μM dose of INA (Figure 1C, detection cutoff at Ct = 33). Similar results were observed in three repetitive experiments. Next, we investigated, if treatment with INA has any adverse effect on the antigenicity of CHIKV by western blot analysis. The binding capacity of the virus to antibody appears to be reduced in INA-inactivated CHIKV virus samples (Figure 1D).

Our findings are important as CHIKV causes an ongoing outbreak affecting millions of people around the globe. An effective CHIKV vaccine is urgently needed to control the spread of CHIKV. Live attenuated CHIKV181/25 was shown to have an excellent immunogenicity; however, the clinical trial was halted due to issues with residual virulence causing transient arthralgia in the vaccine recipients [7]. The INA-inactivated CHIKV181/25 formulation may address the issue of residual virulence that is associated with live attenuated CHIKV181/25. INA-inactivation resulted in a relatively less binding capacity of CHIKV181/25 to the neutralizing polyclonal anti-CHIKV E2 glycoprotein. This raises the question whether there may be some loss of antigenicity upon inactivation with INA. However, INA-inactivated CHIKV may still elicit a protective response against virulent CHIKV infection as shown with other INA-inactivated enveloped viruses such as VEEV, Ebola and Influenza virus. Chemical inactivation of CHIKV for vaccine purposes has been previously reported [11,19]. Formalin inactivated CHIKV was shown to induce neutralizing antibody and reduce homologous virulent virus titer [19]. Further testing of INA-inactivated CHIKV in the animal model will be needed for testing the protective efficacy of INA-inactivated CHIKV181/25 as a vaccine candidate, and its advantage over current approaches of generating inactivated vaccines. To our knowledge this is the first study to show a complete inactivation of CHIKV181/25 using INA.

## Abbreviations

CHIKV: Chikungunya virus; INA: 1, 5 iodonapthyl azide; BHK cells: Baby hamster kidney cells; CPE: Cytopathic effect; DAPI: 4’,6-diamidino-2-phenylindole; PBS: Phosphate buffered saline; BSA: Bovine serum albumin; RT-PCR: Reverse Transcription-Polymerase chain reaction.

## Competing interests

The authors declare that they have no competing interests.

## Authors’ contribution

AS and RKM were involved in the conceptualization and designing of the study. AS carried out the inactivation experiment and analyzed the experimental data. PG carried out the western blot experiments. AS, RKM and PG participated in writing the manuscript. All authors read and approved the final manuscript.
